# The long non-coding RNA LNC_000397 negatively regulates PRRSV replication through induction of interferon-stimulated genes

**DOI:** 10.1186/s12985-022-01761-x

**Published:** 2022-03-05

**Authors:** Jing Zhang, Lipeng Gan, Pu Sun, Jian Wang, Dong Li, Yimei Cao, Yuanfang Fu, Pinghua Li, Xingwen Bai, Kun Li, Xueqing Ma, Huifang Bao, Yingli Chen, Jie Zhang, Zaixin Liu, Zengjun Lu

**Affiliations:** State Key Laboratory of Veterinary Etiological Biology, OIE/National Foot-and-Mouth Disease Reference Laboratory of China, Lanzhou Veterinary Research Institute, Chinese Academy of Agricultural Sciences, Xujiaping No.1, Yanchangpu, Lanzhou, 730046 Gansu China

**Keywords:** PRRSV, lncRNA, RNA-Sequencing, Interferon, Antiviral

## Abstract

**Background:**

Porcine reproductive and respiratory syndrome virus (PRRSV) is one of the most significant threats to the global swine industry. It is of great importance to understand viral-host interactions to develop novel antiviral strategies. Long non-coding RNAs (lncRNAs) have emerged as critical factors regulating host antiviral immune responses. However, lncRNAs participating in virus-host interactions during PRRSV infection remain largely unexplored.

**Method:**

RNA transcripts of porcine alveolar macrophages (PAMs) infected with two different PRRSV strains, GSWW/2015 and VR2332, at 24 h post-infection were sequenced by high-throughput sequencing. Four programs namely, CNCI, CPC, PFAM, and phyloCSF, were utilized to predict the coding potential of transcripts. mRNAs co-localized or co-expressed with differentially expressed lncRNAs were considered as their targets. Fuction of lncRNAs was predicted by GO and KEGG analysis of their target mRNAs. The effect of LNC_000397 on PRRSV replication was validated by knockdown its expression using siRNA. Target genes of LNC_000397 were identified by RNA-Sequencing and validated by RT-qPCR.

**Result:**

In this study, we analyzed lncRNA and mRNA expression profiles of PRRSV GSWW/2015 and VR2332 infected porcine alveolar macrophages. A total of 1,147 novel lncRNAs were characterized, and 293 lncRNAs were differentially expressed. mRNAs co-localized and co-expressed with lncRNAs were enriched in pathogen-infection-related biological processes such as Influenza A and Herpes simplex infection. Functional analysis revealed the lncRNA, LNC_000397, which was up-regulated by PRRSV infection, negatively regulated PRRSV replication. Knockdown of LNC_000397 significantly impaired expression of antiviral ISGs such as MX dynamin-like GTPase 1 (MX1), ISG15 Ubiquitin-like modifier (ISG15), and radical S-adenosyl methionine domain containing 2 (RSAD2).

**Conclusions:**

LNC_000397 negatively regulated PRRSV replication by inducing interferon-stimulated genes (ISGs) expression. Our study is the first report unveiling the role of host lncRNA in regulating PRRSV replication, which might be beneficial for the development of novel antiviral therapeutics.

**Supplementary Information:**

The online version contains supplementary material available at 10.1186/s12985-022-01761-x.

## Introduction

Porcine reproductive and respiratory syndrome (PRRS) is one of the most severe swine diseases worldwide. It is characterized by reproductive failures in sows and respiratory illness in growing pigs, which has caused an enormous economic burden on the swine industry globally. The etiological agent, Porcine reproductive and respiratory syndrome virus (PRRSV), is an enveloped, positive single-stranded RNA virus that belongs to the family Arteriviridae of the order Nidovirales [21]. The genome size of PRRSV varies from 14.9 to 15.5 kb in length, with 11 open reading frames (ORFs). ORF1a and ORF1b, which occupy about 70% of the viral genome, encode two long polyproteins, pp1a and pp1ab. Subsequently, proteolytic processing of these polyproteins yields at least 14 non-structural proteins (nsps), which include four proteases (NSP1α, NSP1β, NSP2, and NSP4), the RNA-dependent RNA polymerase (NSP9), a helicase (NSP10), and an endonuclease (NSP11). ORFs 2–7 encode structure proteins including, glycosylated membrane proteins GP2-GP5, a non-glycosylated membrane protein (M), and the nucleocapsid (N) protein. [5].

The virus was initially isolated in Europe and the United States in the early 1990s and categorized into the European PRRSV and the North American PRRSV genotypes [28]. In 2006, highly pathogenic PRRSV (HP-PRRSV), a novel variant of type 2 PRRSV featured by the 30-aa deletion in the coding region of non-structure protein 2, emerged in China [12]. The clinical features of HP-PRRSV infections are high fever, high morbidity, and fatality. Since its first outbreak, HP-PRRSV has caused an inestimable economic loss. A novel NADC30-like virus with 133-aa deletion in the nsp2 was isolated in the field in 2013 [36]. It has been reported that NADC30-like viruses are undergoing recombination with HP-PRRSV, which makes disease control more challenging.

PRRSV infection impairs host innate and adaptive immune responses, leading to ineffective virus clearance and the establishment of chronic infection. The type I interferon response is a critical component of the host innate immune response against virus infection. The receptor for type I IFNs is ubiquitously expressed. Upon IFN binding, transmits the signal through two receptor-associated tyrosine kinases, tyrosine kinase 2 (TYK2) and Janus kinase 1 (JAK1), resulting in the phosphorylation of signal transducers and activators of transcription STAT1 and STAT2. The phosphorylated STATs form heterodimers and bind to IFN-regulatory factor 9 (IRF9) to form the active transcription factor complex IFN-stimulated gene factor 3 (ISGF3). ISGF3 initiates transcription by binding to the promoters of interferon-stimulated genes (ISGs), which contain IFN-stimulated response elements (ISREs) [26, 27]. Some ISGs encode proteins with direct antiviral activity, such as ISG15, Mx1, Viperin, and interferon-inducible RNA-dependent protein kinase(PKR). Many of the ISGs encode transcription factors that enhance the production of interferons and other cytokines.

PRRSV has developed a set of mechanisms for suppressing interferon signaling. Several viral proteins have been reported to impair IFN production (Nsp1, Nsp2, Nsp4, Nsp11, and N protein) [22]. Nsp1 translocates to the nucleus, inhibits the binding of transcription factor IRF3 with CBP, and facilities CBP degradation, resulting in down-regulation of IFN promoter activity [8]. Nsp1β restrains STAT1 phosphorylation and ISGF3 nuclear translocation. Nsp2 inhibits NF-κB activation by interfering with the polyubiquitination process of IκBα, subsequently preventing the degradation of the IκBα protein, which are required for the releasing of NF-κB dimers and their translocation to the nucleus, where they regulate transcription of type I interferons [23]. Nsp11 induces STAT2 degradation, and it also interacts with IRF9 to impair the nuclear translocation of the ISGF3 complex [33]. Nsp11 and N protein both inhibits IRF3 phosphorylation and nuclear translocation to prevent IFN transcription [19]. N protein also interacts with SOCS1 (a negative regulator of JAK-STAT signaling) to inhibit IFN production [14].

Non-coding RNAs include long ncRNAs (lncRNAs) and short ncRNAs such as microRNAs (miRNAs), PIWI-interacting RNAs (piRNAs), and small nuclear RNAs (snRNAs) [6]. By definition, lncRNAs are transcripts lacking protein-coding potential and longer than 200 nt. The majority of lncRNAs are 7-methylguanosine-capped, spliced, and polyadenylated. A number of reports have delineated the critical roles of lncRNAs in a wide variety of biological processes [15]. Precise regulation of gene expression in the immune system is crucial to protect hosts from pathogen infections by generating effective immune responses while limiting autoimmunity [20]. Emerging evidence has established that lncRNAs are involved in the regulation of immune cell differentiation and immune responses. LncRNAs modify the virus-host interactions by regulating ISG expression or through ISG independent manner, such as stabilizing virus RNA structure or modulating viral replication [17, 18, 25–27].

The role of host proteins and microRNAs in virus-host interactions of PRRSV has been extensively studied. However, the function of host lncRNAs in regulating PRRSV-induced immune response is largely unknown. Transcriptomic profiles of PRRSV infected host cells have been analyzed either in vitro or in vivo [1, 29, 31]. These studies revealed that PRRSV infection resulted in alteration of gene expression evolved in innate immune response signaling pathways such as type I IFN signaling, TLR, and RIG-I signaling. MicroRNAs also play critical roles in regulating host innate immunity. For example, miR-24-3p facilitates PRRSV replication by suppression of HO-1 expression [31]. MiR-373 promotes PRRSV replication through impairing type I IFN production [4]. However, the functions of lncRNAs in host immune response against PRRSV are unknown. In our previous study, we analyzed expression profiles of HP-PRRSV strain GSWW/2015 and FL-12 infected PAMs by RNA-sequencing to identify lncRNAs regulated by PRRSV infection. We found a lncRNA, TCONS_00054158, induced by different strains of PRRSV and poly (I: C) but not by the heat-inactivated virus. The function of this lncRNA is under study in our lab (Zhang et al., 2017b). Zhen and colleagues analyzed mRNA, lncRNA, and microRNA profiles of PRRSV infected PAMs from two pig breeds [35]. Another study used co-expression network analysis of differently expressed mRNAs and lncRNAs after PRRSV infection to identify the function of these lncRNAs. They found some of the lncRNAs associated with the interferon-induced genes [30].

To explore the role of lncRNAs in the virus-host interaction of PRRSV, we sequenced RNA transcripts of PAMs infected with GSWW/2015 (GSWW for short) and vaccine strain VR2332 at 24 h post-infection. Using rigorous methods to evaluate the coding potential of the transcripts, 1,147 novel lncRNAs were characterized, and a total of 293 lncRNAs were differentially expressed. We identified a lncRNA, LNC_000397, which was up-regulated after PRRSV infection and negatively regulated PRRSV replication by inducing ISGs. This research firstly reported the function of lncRNA in regulating PRRSV replication, which might be beneficial for understanding the interaction between PRRSV and the host immune system.

## Material and methods

### Cells and viruses

PAMs were isolated from the lung lavage fluid of 4-week-old SPF (Specific Pathogen-Free) pigs as described previously [34]. PAMs were maintained in RPMI-1640 medium, and Marc-145 cells were cultured in DMEM. Both culture media were supplemented with 10% heat-inactivated FBS (GIBCO), 100 U/ml penicillin, and 100 mg/ml streptomycin. Cells were incubated at 37℃ in a humidified atmosphere of 5% CO_2_. GSWW/2015 (GenBank accession number: KX767091.1, https://www.ncbi.nlm.nih.gov/nuccore/KX767091) was isolated by our laboratory and characterized as an HP-PRRSV strain. These PRRSV strains were propagated in Marc-145 cells, and viral titers were determined using TCID_50_ assay.

### RNA isolation, reverse transcription, and qPCR

Total RNA was isolated using the RNeasy kit (Qiagen) according to the manufacturer’s protocol. Equal amounts of total RNA were reverse transcribed using the PrimeScript™ RT Master Mix (Takara). cDNA was then subjected to qPCR using TB Green® Premix Ex Taq™ II (Takara) on a QuantStudio 5 Real-Time PCR system (Applied Biosystems). Relative expression of lncRNAs and mRNAs was calculated using the comparative Ct method as previously described [34]. Primers for qPCR are listed in Additional file 8: Table S7.

### RNA-sequencing (sample preparation, library construction, and sequencing)

A total of 1 × 10^7^ PAMs were seeded in a 25 cm^2^ flask and cultured for 12 h before virus infection. Then PAMs were mock-infected or infected with GSWW and VR2332 at an MOI of 0.1 for 24 h. Each group contains three biological repeats. Subsequently, cells were washed with PBS once and harvested. Total RNA was extracted using TRizol (Thermo Fisher) following the manufacturer’s instruction. RNA concentration and purity were measured by Nanodrop 2000, and RNA integrity was assessed by the Bioanalyzer 2100 system (Agilent Technologies). In all, 3 µg RNA per sample was used as input for the RNA-sequencing library construction. The procedure was conducted as previously described [34]. Briefly, ribosomal RNA was removed, and residual RNAs were cleaned using ethanol precipitation. Nine sequencing libraries were generated using the NEBNext® Ultra™ Directional RNA Library Prep Kit for Illumina® (NEB, USA). The quality of the libraries was checked on an Agilent Bioanalyser 2100 system. The libraries were sequenced on an Illumina Hiseq X TEN platform, and 150 bp paired-end reads were generated. The RNA-Seq and data collection were performed by Novogene Co. LTD, Beijing, China.

### Data analysis of RNA-sequencing

Raw data of fastq format were firstly processed by in-house Perl scripts of Novogen. Clean data were acquired by removing adapter sequence, reads containing poly-N, and low-quality reads from raw data. Meanwhile, Q20, Q30, and GC content of the clean data were calculated. All the downstream analyses were based on clean data with high quality (Q30 > 90%). The paired-end clean reads were aligned to the porcine reference genome (Sscrofa11.1) by TopHat v2.0.9. The mapped reads of each sample were assembled by Cufflinks (v2.1.1).

### LncRNA identification

To identify lncRNAs, the assembled transcripts were first filtered by FPKM > 0.5, coverage > 1, and length > 200 nt to remove background noise. Subsequently, four programs namely, CNCI (Coding-Non-Coding-Index), CPC (Coding Potential Calculator), Pfam, and phyloCSF (phylogenetic codon substitution frequency), were utilized to analyze the coding potential of filtered transcripts. CNCI profiles adjoining nucleotide triplets to discriminate protein-coding and non-coding sequences independent of known annotations. CPC (0.9-r2) mainly evaluates the extent and quality of the ORF in a transcript and searches the sequences with known protein sequence databases to distinguish the coding and non-coding transcripts [9]. Each transcript was translated in all three possible frames, and Pfam Scan was used to find the occurrence of any of the known protein family domains documented in the Pfam database [16]. Transcripts with a Pfam hit would be excluded in the following steps [2]. PhyloCSF scans evolutionary characteristics to alignments of conserved coding regions to clarify protein-coding and non-coding transcripts [13]. Transcripts predicted with coding potential by either of the four programs above were excluded.

### Differential gene expression analysis of RNA-Sequencing data

The FPKM (fragments per kilobase per million reads) values of both lncRNAs and mRNAs in each sample were computed by Cuffdiff (v2.1.1) [24]. Gene expression levels were calculated by summing the FPKMs of transcripts in each gene group. Cuffdiff provides statistical routines for determining differential expression in gene expression data by a model based on the negative binomial distribution [24]. Transcripts with a *P*-value < 0.05 were considered as differentially expressed genes.

### LncRNA target gene prediction

Most of the porcine lncRNAs have not yet been functionally annotated. In this study, the prediction of their target genes is based on the functional annotations of their related *cis* and *trans* target mRNAs. Coding genes located within 100 kb upstream and downstream of lncRNAs in the genome were searched and assigned as lncRNA co-location genes (cis-activating lncRNAs). The expressed correlations between lncRNAs and coding genes were calculated with custom scripts. Coding genes and lncRNAs with Pearson correlation score > 0.95 or < − 0.95 were considered co-expressed (trans-activating lncRNAs). The functions of lncRNAs were predicted by functional enrichment analysis of their co-location and co-expressed coding genes.

### GO and KEGG enrichment analysis of RNA-sequencing data

Gene Ontology (GO) enrichment analysis of differently expressed mRNAs and lncRNA target genes were executed by the GOseq R package. GO terms with corrected *P*-value < 0.05 were considered significantly enriched. KOBAS software was utilized to test the statistical enrichment of DE mRNAs and lncRNA target genes in KEGG pathways (Kyoto Encyclopedia of Genes and Genomes) [32].

### siRNA and cell transfection

The siRNAs were purchased from GenePharma (China) and JTS BIO (China). The sequences of siRNAs are listed in Additional file 8: Table S7. siRNAs were transiently transfected with Lipofectamine 3000 (Thermo Fisher) according to the manufacturer's instructions. The amount of siRNA used for transfection was optimized to avoid cellular toxicity, and 50 nM siRNA was used.

## Results

### Identification of DE lncRNAs after PRRSV infection by RNA-sequencing

To identify PRRSV-regulated lncRNAs in porcine primary alveolar macrophages, we performed RNA-Sequencing of total RNA without rRNA extracted from PRRSV-infected PAMs. Specifically, PAMs isolated from three 4-week-old SPF pigs were infected with GSWW, VR2332 at an MOI of 0.1, or mock-infected were collected at 24 hpi. Samples were prepared from triplicates and nine sequencing libraries were constructed. Approximately 108 million (range from 91.6 to 119 million) 150 bp paired-end clean reads were generated on average of each sample after removing adapter fragments and low-quality reads. Clean reads were aligned to Sus Scrofa (sscrofa 11.1) genome with TopHat. Transcripts were assembled using Cufflinks. The expression levels of transcripts were calculated by cuffdiff and shown as fragments per kilobase per million reads (FPKMs).

**Fig. 1:**
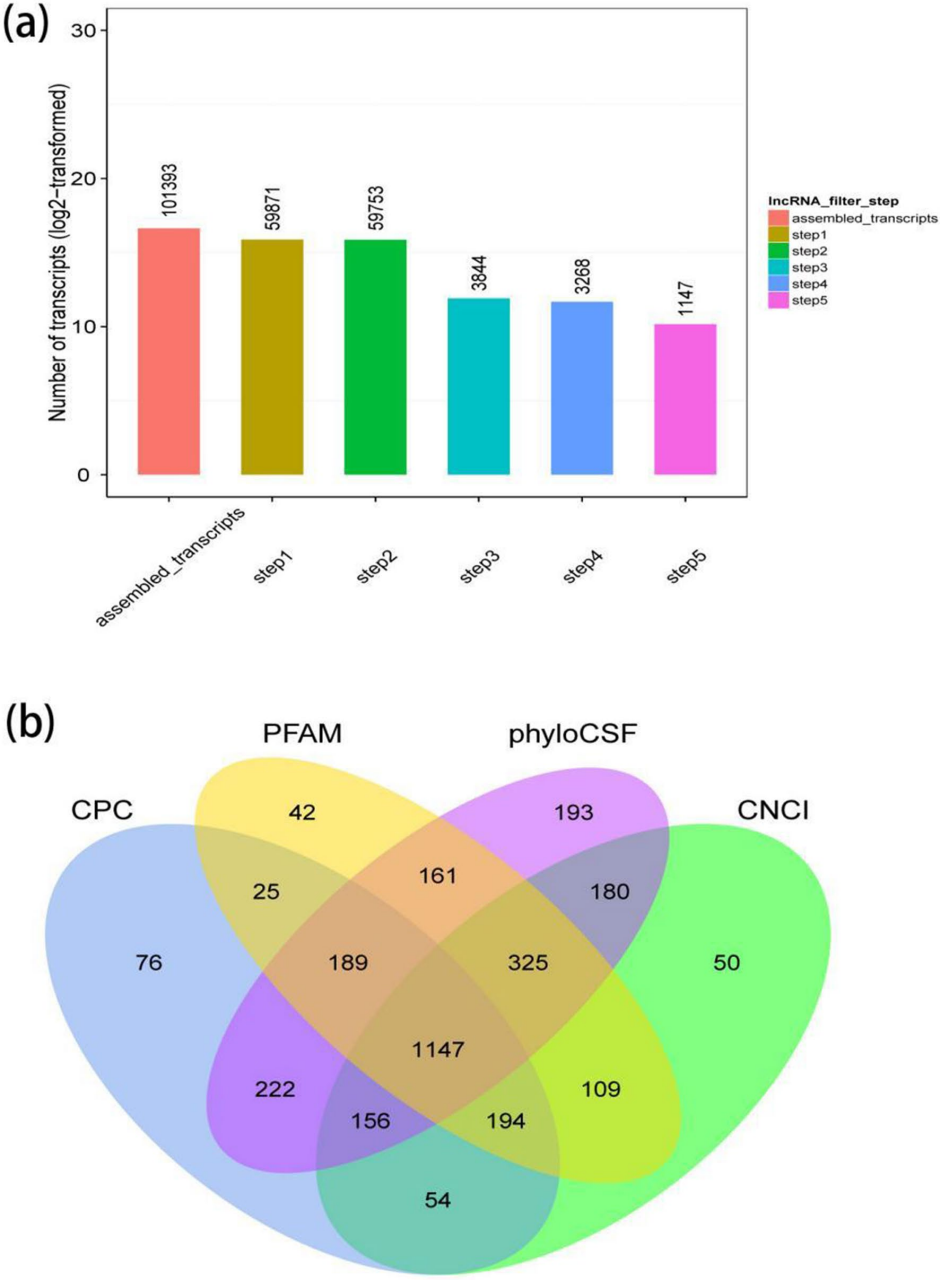
**a** Five steps were performed to predict novel lncRNAs. The X-axis stood for each step, and Y-axis stood for the number of transcripts left after each step. First, single-exon transcripts were filtered out. Second, transcripts longer than 200 nt were kept. Third, using Cuffcompare, transcripts overlapped with exons of annotated mRNAs were assigned as annotated lncRNAs. Fourth, transcripts expressed at low levels (FPKM < 0.5) were removed. Fifth, four programs were applied to predict the coding potential of transcripts, namely CNCI, CPC, Pfam-scan, and phyloCSF. **b** The Venn diagram showed numbers of predicted non-coding transcripts by CNCI, CPC, Pfam-scan, and phyloCSF. A total of 1147 transcripts predicted as non-coding transcripts by all the algorithms were characterized as novel lncRNAs

As shown in Fig. 1a, five steps were performed to identify novel lncRNAs. First, single-exon transcripts, which might be transcription noise, were filtered out. Second, transcripts longer than 200 bp were kept according to the definition of lncRNA. Third, Cuffcompare was utilized to identify transcripts overlapped with exons of annotated mRNAs, which were assigned as annotated lncRNAs. We also explored a domestic-animal long non-coding RNA database (ALDB) to find annotated lncRNAs [10]. Then transcripts expressed at low levels (FPKM < 0.5) were removed. Last, we used CNCI, CPC, Pfam-scan, and phyloCSF to predict the coding potential of transcripts. Transcripts predicted with coding potential by either of the four programs above were filtered out (Fig. 1b). After these steps, 1147 transcripts were predicted as novel lncRNAs.

**Fig. 2:**
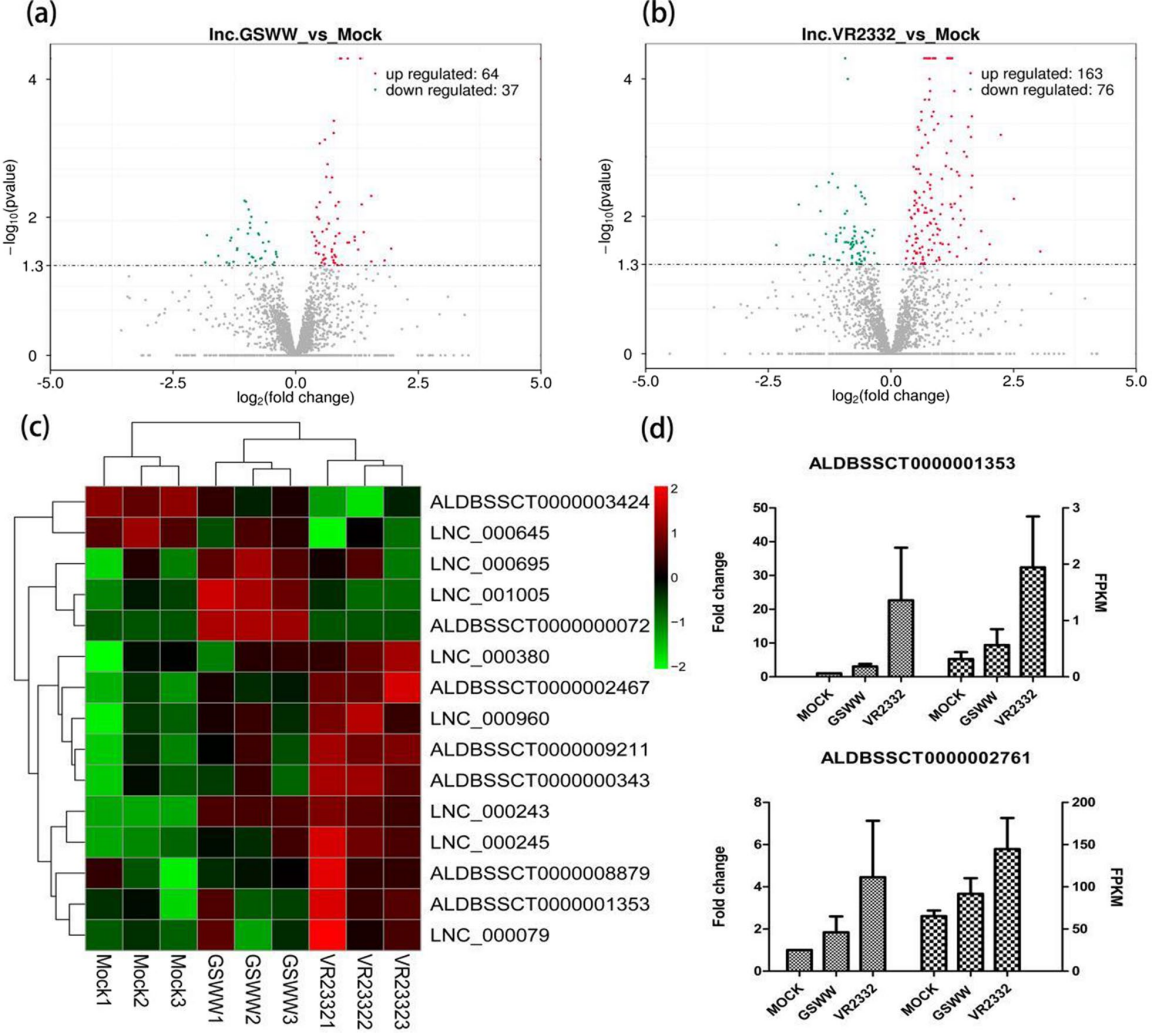
Volcano plots show differentially expressed lncRNAs in **a** GSWW versus Mock comparison group and in **b** VR2332 vs. Mock comparison group. **c** Hierarchical heat map demonstrating transformed expression values (log2 FPKM) for 15 differentially expressed lncRNAs. Red shows up-regulation and green shows down-regulation. **d** RT-qPCR results of differentially expressed genes after PRRSV infection by GSWW and VR2332 at 24 hpi in PAMs. Total RNA was extracted, and the first-strand cDNA was synthesized using reverse transcriptase kit. The bar represents the mean of three samples. Expression levels were normalized to GAPDH

Using cuffdiff, a total of 441 and 1509 differently expressed mRNAs (DE mRNAs) were identified between the GSWW and VR2332 groups, respectively (Additional file 2: Table S1). The numbers of differently expressed lncRNAs (DE lncRNAs) were less than mRNAs in both comparison groups, which was 101 in GSWW and 239 in the VR2332 infection group, respectively (Fig. 2a, b, and Additional file 3: Table S2). Figure 2c showed a heat map of 15 DE lncRNAs after PRRSV infection. The data demonstrated hierarchical clustering of log2 (FPKM) at each sample. We selected ten genes to validate their expression by RT-qPCR (Fig. 2d and Additional file 1: Figure S1). Five mRNAs and five lncRNAs were tested, the correlation between the RT-qPCR results and RNA-Seq data was measured by Pearson’s correlation coefficient. A strong correlation between the two methods was observed for all the tested genes (Table 1).

**Table 1:** Correlation analysis of RNA sequencing and RT-qPCR results

Transcript name		MOCK	GSWW	VR2332	Correlation score
ALDBSSCT0000001353	FPKM	0.317347	0.564727	1.943572	0.998
	RT-QPCR	1	3.053109	22.66456	
CD274	FPKM	16.00107	25.69197	39.9606	0.998
	RT-QPCR	1	1.771723	3.207748	
TNFSF10	FPKM	22.69663	44.223	97.9314	0.994
	RT-QPCR	1	2.105087	7.100317	
LNC_000397	FPKM	0.517206	0.885413	1.229253	1.000
	RT-QPCR	1	1.510961	2.025578	
ALDBSSCT0000002761	FPKM	65.2994	91.73363	144.6964	0.995
	RT-QPCR	1	1.840139	4.459228	
RNASEL	FPKM	4.922467	9.364923	21.53297	0.997
	RT-QPCR	1	1.221049	1.654972	
CD169	FPKM	5.571897	14.4758	42.70057	0.997
	RT-QPCR	1	1.715125	5.186836	
PKR	FPKM	17.13503	42.66707	63.8304	1.000
	RT-QPCR	1	2.667194	4.195808	
LNC_000695	FPKM	7.480513	15.6161	10.31643	0.999
	RT-QPCR	1	1.562077	1.169119	
LNC_000454	FPKM	0.699077667	1.057696667	1.074059667	0.886
	RT-QPCR	1	1.306818	1.174907	

### Prediction of lncRNA targets

LncRNAs regulate the expression of their target mRNA through various mechanisms. It has been reported that lncRNAs could regulate the transcription of their neighboring protein-coding genes (cis-acting lncRNAs). We predicted the function of lncRNAs by analyzing protein-coding genes located within 100 kb upstream and downstream of lncRNAs in the genome (Additional file 6: Table S5). The mRNAs showed correlated expression patterns with differently expressed lncRNAs were more likely to be modulated by the lncRNAs (trans-acting lncRNAs). Therefore, we used Pearson correlation analysis to identify co-expressed lncRNAs and mRNAs in each comparison group. The lncRNA-mRNA pairs with Pearson correlation score > 0.95 or < − 0.95 were considered co-expression (Additional file 7: Table S6).

### GO enrichment and KEGG pathway analysis of DE mRNAs and predicted lncRNA targets

To characterize PRRSV-infection-induced functional alterations of RNA expression profiles, we performed Gene Ontology enrichment analysis of DE mRNAs. Up-regulated genes were enriched in 33 and 16 biological processes upon GSWW or VR2332 infection, respectively. Most of them were related to virus infection and immune response such as immune effector process, response to virus, and defense response to virus. (Additional file 1: Figure S2 and S Additional file 4: Table S3). We also performed KEGG enrichment analysis of DE mRNAs to identify pathways involved in PRRSV infection. We found that up-regulated mRNAs of both groups were enriched in virus infection-related pathways such as Influenza A and Herpes simplex infection. DE mRNAs in the GSWW infection group were enriched in innate immune response pathways such as Toll-like receptor, RIG-I-like receptor, and NF-κB signaling pathway (Additional file 1: Figures S3–S5 and Additional file 5: Table S4).

**Fig. 3:**
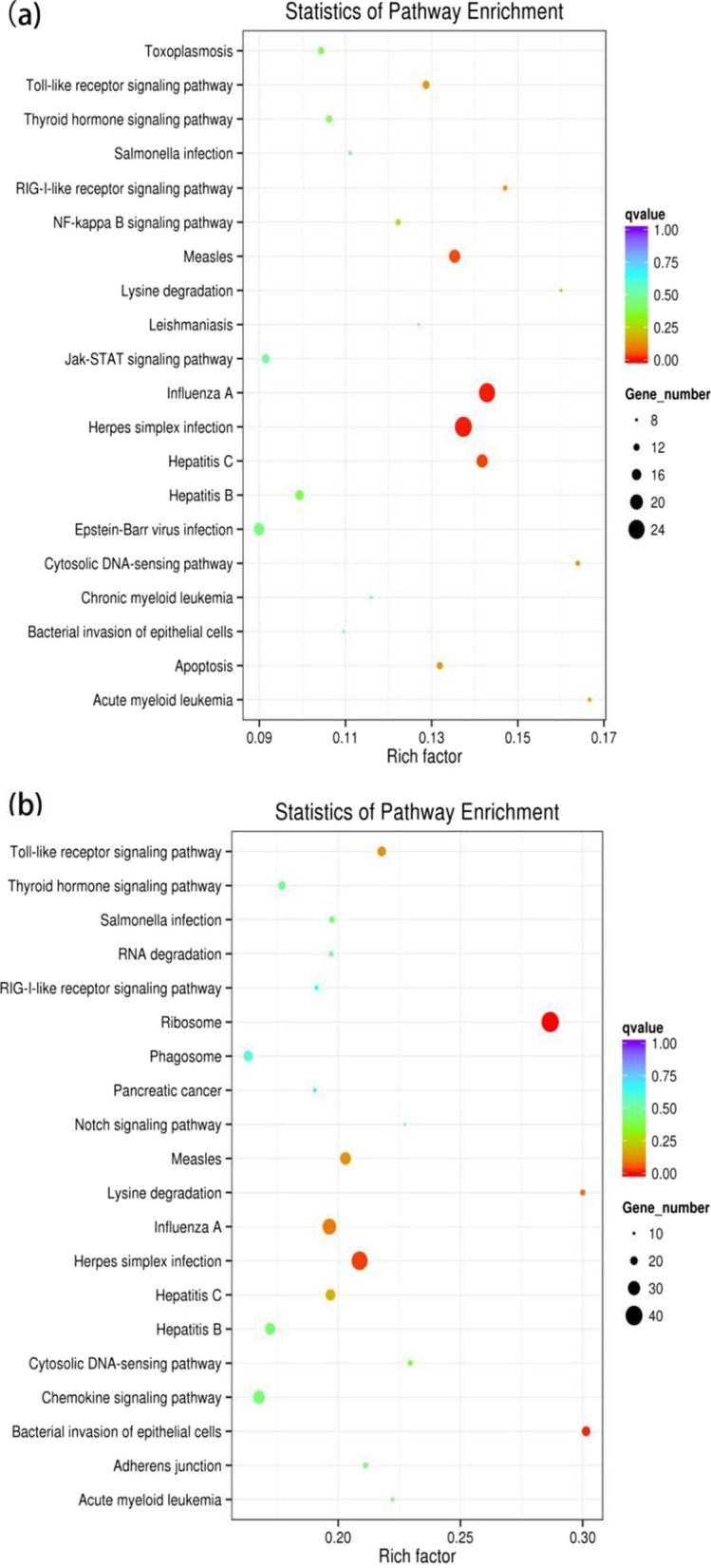
Scatter plots of KEGG pathway enrichment statistics. Top 20 statistics of pathways, enrichment in the KEGG database for neighboring mRNAs of differentially expressed lncRNAs in **a** the GSWW infection group and the **b** VR2332 infection group

**Fig. 4:**
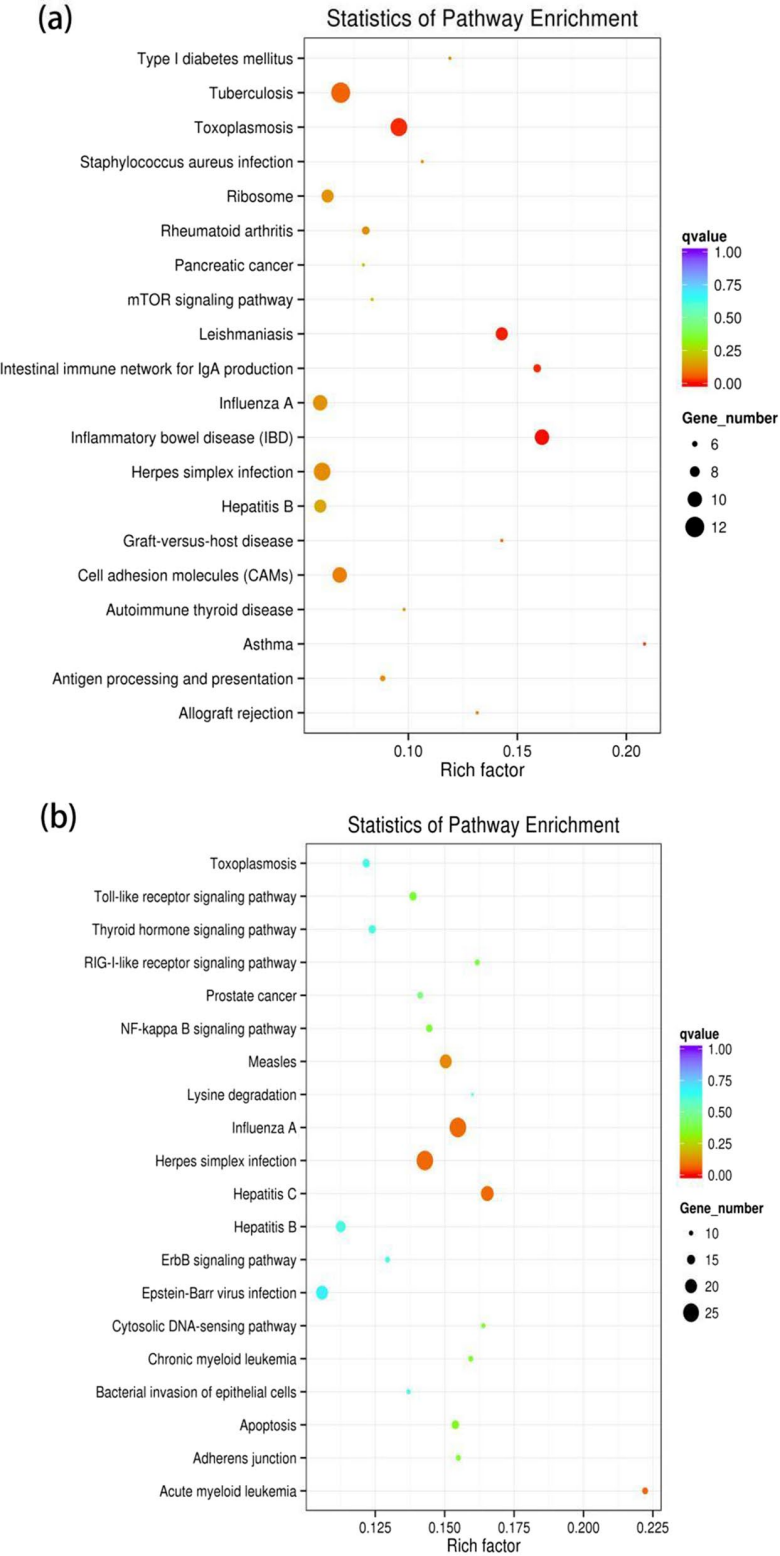
Scatter plots of KEGG pathway enrichment statistics. Top 20 statistics of pathways, enrichment in the KEGG database for co-expressed mRNAs of differentially expressed lncRNAs in **a** the GSWW infection group and **b** the VR2332 infection group

Due to the lacking of functional annotation of porcine lncRNAs, KEGG analysis of co-localized and co-expressed mRNAs was utilized to predict lncRNA function. KEGG analyses of mRNAs co-localized with DE lncRNAs (cis-acting lncRNAs) in the GSWW infection group were enriched in immune response-related pathways such as Inflammatory bowel disease, Leishmainasis, Intestinal immune network for IgA production, and so on (Fig. 3a). There was no significantly enriched pathway of mRNAs co-localized with up-regulated lncRNAs in the VR2332 infection group. We only identified one pathway enriched in mRNAs co-localized with down-regulated lncRNAs in the VR2332 infection group, which was Inflammatory bowel disease (Fig. 3b). As for the functional prediction of trans-activating lncRNAs, we found that mRNAs co-expressed with up-regulated lncRNAs were enriched in virus infection-related pathways such as Herpes simplex infection in both groups (Fig. 4 and Additional file 7: Table S6). We did not identify significantly enriched pathways of mRNAs co-expressed with down-regulated lncRNAs in both groups.

### Validation of lncRNAs function

**Fig. 5:**
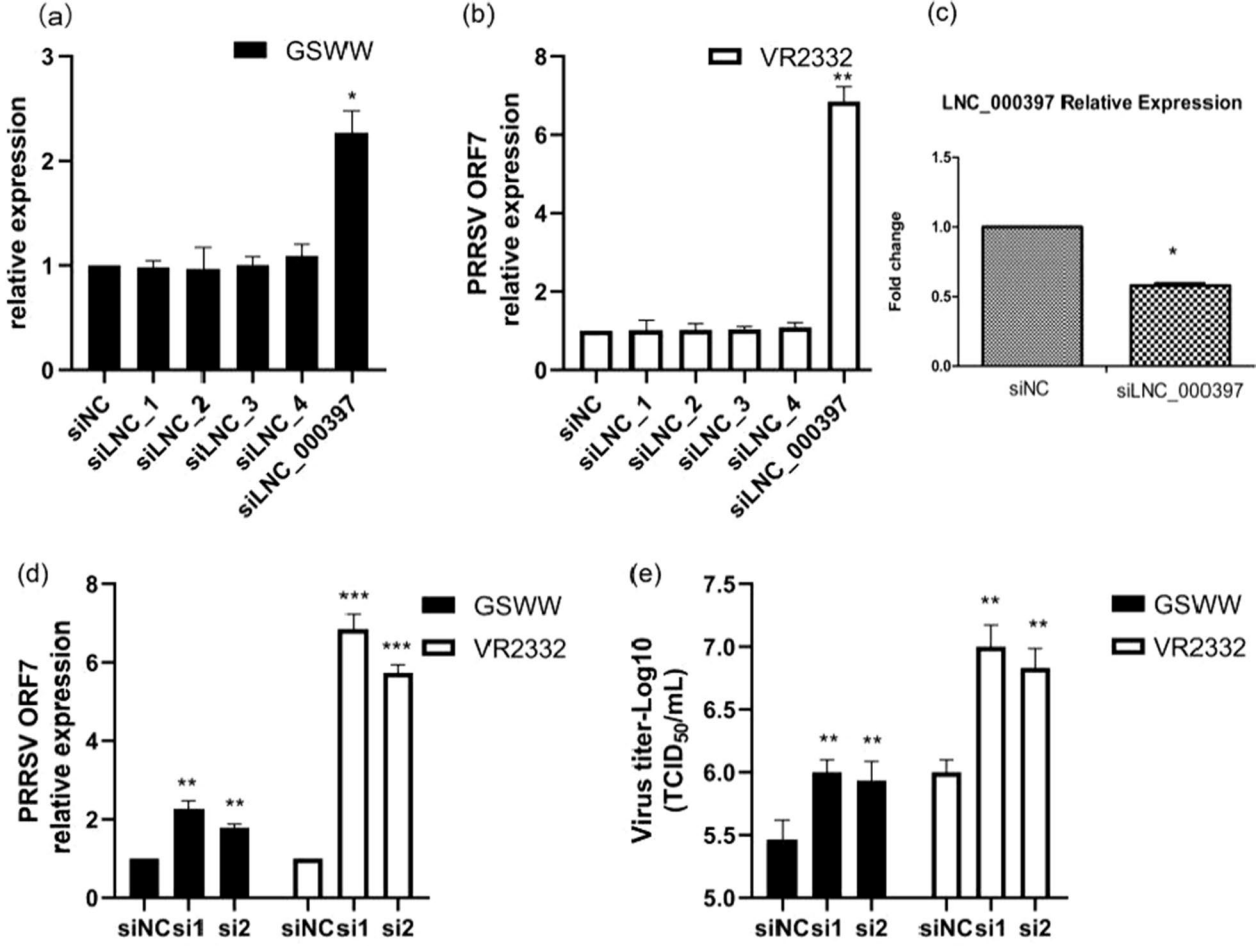
**a** and **b** Screening of lncRNAs that regulate PRRSV replication. PAMs were transfected with siRNAs targeting five up-regulated lncRNAs, 24 h after transfection cells were inoculated with **a** GSWW or **b** VR2332 with an MOI of 0.1. At 24 hpi, total RNAs were subjected to qRT-PCR analysis for PRRSV ORF7. **c** The knockdown efficiency of LNC_0003997 was determined by qRT-PCR. PAMs were transfected with 50 nM Negative Control (NC) siRNA or two distinct siRNAs against LNC_000397 for 24 h before infecting them with GSWW or VR2332 (MOI = 0.1). At 24 hpi, total RNAs were extracted for PRRSV ORF7 quantification using qRT-PCR. **d** and the supernatants were collected for TCID50 determination (**e**). The bar represents the mean of three independent experiments. Expression levels were normalized to GAPDH

To further explore the function of these DE lncRNAs, we chose five up-regulated lncRNAs to study. Since it is technically challenging to force the expression of a foreign gene in PAMs, we reduced their transcription by transfecting siRNAs targeting these lncRNAs. After transfection, cells were infected with GSWW and VR2332, and virus copies numbers were analyzed by RT-PCR at 24 hpi (Fig. 5a and b). Knockdown of LNC_000397 increased PRRSV RNA copy numbers, about twofold by GSWW infection and sixfold by VR2332 infecton (Fig. 5c and d). To validate the role of LNC_000397 in suppressing PRRSV replication, we also used TCID_50_ assay to determine virus titers. The results showed that knockdown of LNC_000397 increased virus titers on Marc-145 cells, about fivefold by GSWW infection and tenfold by VR2332 infection (Fig. 5e). LNC_000397 locates at chromosome 13, whose full length is 8540 nt. We cloned the full length of this lncRNA from PAMs and sent for sanger-sequencing. The sequence was identical to that got from RNA-Seq.

**Fig. 6:**
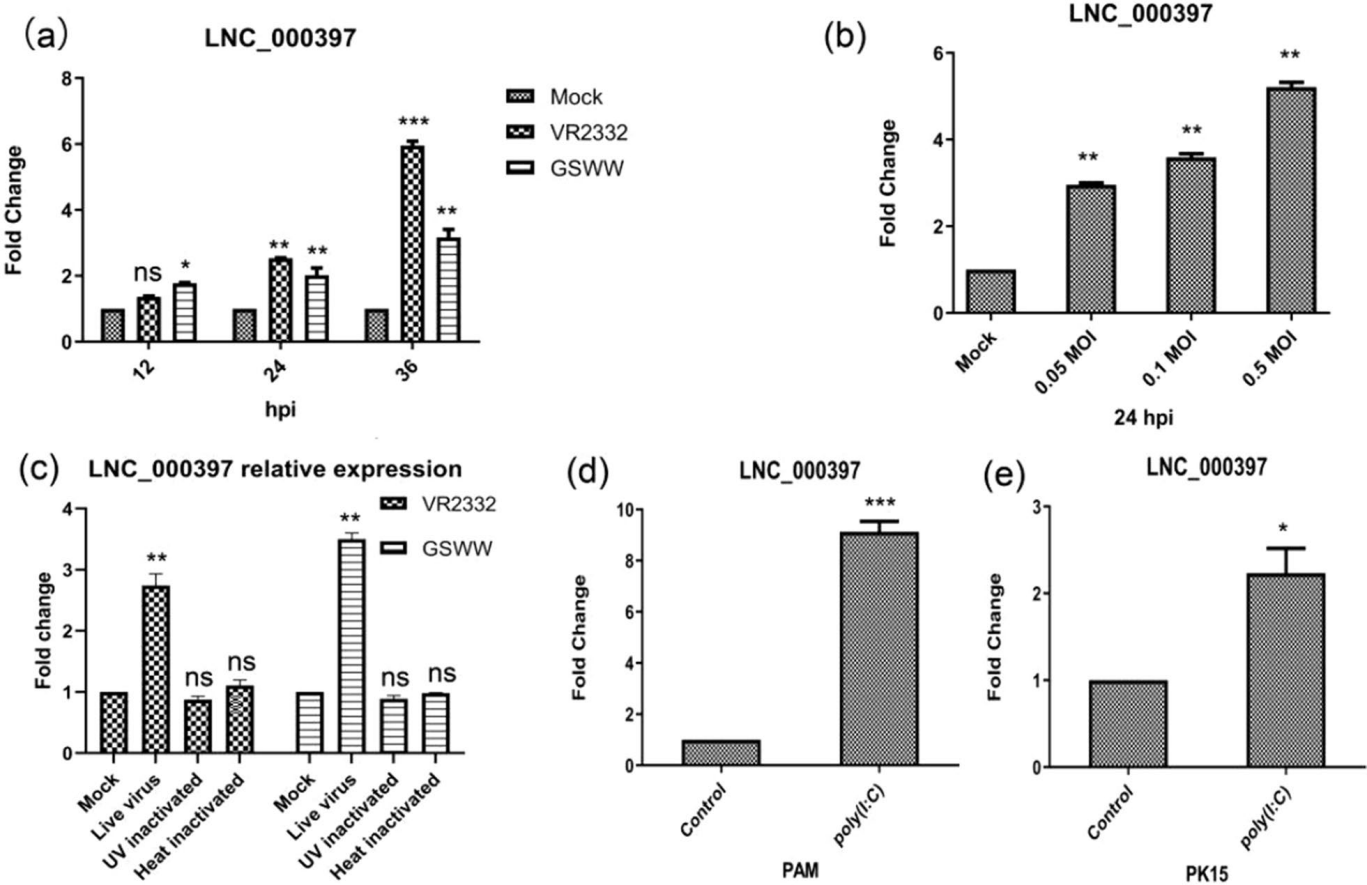
**a** PAMs were either mock-infected or infected with GSWW or VR2332 (MOI = 0.1). At 12, 24 and 36 hpi, the LNC_000397 levels were determined by qRT-PCR. **b** PAMs were either mock-infected or infected with the VR2332 at MOIs of 0.05, 0.1, 0.5. At 24 hpi, the LNC_000397 mRNA levels were determined by qRT-PCR. **c** PAMs were mock-infected or infected with GSWW or VR2332 (MOI = 0. 1), UV-inactivated, heat-inactivated GSWW, or VR2332. LNC_000397 were quantified by qRT-PCR at 24 hpi. **d** and **e** PAMs and PK-15 cells were transfected with 2 μg/ml poly(I: C), 24 h after transfection, the LNC_000397 mRNA levels were determined by qRT-PCR. The bar represents the mean of three independent experiments. Expression levels were normalized to GAPDH

Then we inoculated PAMs with GSWW and VR2332 strains at an MOI of 0.1 and found that the LNC_000397 expression level was increased from 12 hpi in a time-dependent manner, the induction was most obvious at 36 hpi (about sixfold by VR2332 infection and 2.5 fold by GSWW infection) (Fig. 6a). Since VR2332 induced its expression more obviously, we infected PAMs with VR2332 at MOIs of 0.05, 0.1 and 0.5. The results showed that the expression of LNC_000397 was induced in a dose-dependent manner (Fig. 6b). To study whether induction of LNC_000397 depends on virus replication, we treated PAMs with UV-inactivated and heat-inactivated VR2332 and found that inactivated virus was not able to induce LNC_000397 expression (Fig. 6c). Poly(I: C) transfection also increased LNC_000397 expression in PAM (about ninefold) and PK-15 cells (about twofold) (Fig. 6d and e).

**Fig. 7:**
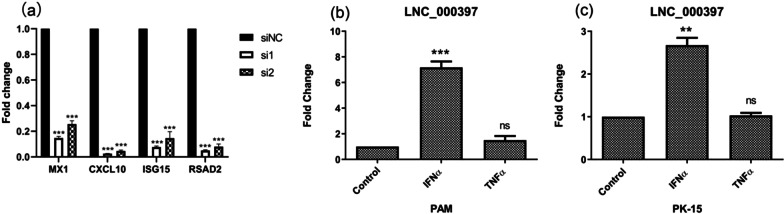
**a** PAMs were transfected with 50 nM NC siRNA or two distinct siRNAs against LNC_000397, 24 h after transfection total RNA was extracted from cell lysates. MX1, CXCL10, ISG15, RSAD2 were quantified by qRT-PCR at the indicated times. **b** PAMs and **c** PK15 cells were treated with IFN-α and TNF-α, 24 h after treatment, total RNAs were extracted and the LNC_000397 mRNA levels were determined by qRT-PCR. The bar represents the mean of three independent experiments. Expression levels were normalized to GAPDH

To study its functional mechanism, we utilized RNA-Seq to identify target genes of Lnc_000397. We found that top genes suppressed by LNC_000397 knockdown were interferon-stimulated genes that play essential roles in antiviral response, such as CXCL10 (C-X-C Motif Chemokine Ligand 10), IFIT2 (Interferon-Induced Protein With Tetratricopeptide Repeats 2), and RSAD2 (Radical S-Adenosyl Methionine Domain Containing 2). We validated the sequencing results by RT-qPCR. Our results showed that knockdown of LNC_000397 decreased more than 70% expression of these ISGs (Fig. 7a). Then we asked whether LNC_000397 expression is induced by interferon. We treated PAMs with IFN-α and TNF-α, the results showed that LNC_000397 expression was induced by IFN-α (about sevenfold in PAM and 2.5 fold-in PK cells) but not TNF-α (Fig. 7b and c). These results indicated that LNC_000397 suppressed PRRSV replication by inducing the expression of ISGs, and it was also up-regulated by interferon treatment.

## Discussion

PRRSV has caused tremendous economic losses to the world swine industry. The high mutation rate and immune suppression nature of the virus make it challenging for vaccine development. Further understanding of the interaction between PRRSV and the host immune system would be beneficial for the implementation of novel antiviral strategies. One of the main goals of this study was to explore the role of host lncRNAs in regulating PRRSV-induced immune response. In this research, we characterized a total of 293 differently expressed lncRNAs after infection with two PRRSV strains. Inconsistent with our and other groups’ reports, predicted targets of DE lncRNAs were enriched in immune-response related pathways such as NF-κB and RIG-I signaling [7, 34].

Importantly, we identified a lncRNA, LNC_000397, suppressed PRRSV replication in PAMs. The expression of lnc_000397 was up-regulated in PRRSV GSWW and VR2332 infected cells. It also could be induced by poly (I: C), a synthetic dsRNA analog, in PAM and PK15 cells. Using RNA-seq, we found that knocking down of this lncRNA down-regulated the expression of ISGs such as CXCL10, MX1, ISG15, and RSAD2. Furthermore, we demonstrated that LNC_000397 was induced by type I-IFN treatment. Because of the low transfection efficiency of primary macrophage, we could not detect the effect of LNC_000397 over-expression on PRRSV replication. Our results suggested that LNC_000397 is an IFN-dependent antiviral lncRNA.

Interferon signaling is one of the most critical parts of innate immunity to defense against virus infection. To date, the vast majority of ISGs involved in the antiviral immune response are proteins, such as Mx, IFIT protein family, and OAS protein family. In recent years, the roles of interferon-dependent lncRNAs have emerged. An IFN-β induced lncRNA, lnc-ISG20, inhibits IAV replication by enhancing ISG20 expression [3]. Lnc-MxA is an interferon-stimulated gene (ISG) functions as a negative regulator of the antiviral immune response [11]. These reports indicated that lncRNAs represent another set of ISGs that exert important roles in the antiviral immune response. Our studies showed that LNC_000397 was stimulated by PRRSV infection and negatively regulated virus replication as a novel ISG.

## Conclusions

Our study has shown that LNC_000397 was up-regulated by PRRSV infection in PAMs and impaired PRRSV replication by up-regulating ISGs expression. This research is the first report revealing the role of a lncRNA in regulating PRRSV replication, which might provide new insights for the development of novel antiviral strategies.

## Supplementary Information

**Additional file 1: Figure S1** RT-qPCR results of eight differentially expressed genes after PRRSV infection by GSWW and VR2332 at 24 hpi. Total RNA was extracted by Trizol, and the first strand cDNA was synthesized using reverse transcriptase kit. Bar represents the mean of three samples. Expression levels were normalized to GAPDH. Figure S2 shows GO enrichment of up-regulated mRNAs in GSWW (a) and VR2332 (b) infected groups. Figure S3 shows Scatter plots of KEGG pathway enrichment statistics. Top 20 statistics of pathways, enrichment in the KEGG database for up-regulated mRNAs in GSWW infection group. Figure S4 shows Scatter plots of KEGG pathway enrichment statistics. Top 20 statistics of pathways, enrichment in the KEGG database for up-regulated mRNAs in VR2332 infection group. Figure S5 shows Scatter plots of KEGG pathway enrichment statistics. Top 20 statistics of pathways, enrichment in the KEGG database for down-regulated mRNAs in VR2332 infection group.

**Additional file 2: Table S1** Lists differently expressed mRNAs upon GSWW and VR2332 infection compared to Mock infection.

**Additional file 3: Table S2** Lists differently expressed lncRNAs upon GSWW and VR2332 infection compared to Mock infection.

**Additional file 4: Table S3** Lists GO enrichment of up-regulated and down-regulated mRNAs in GSWW and VR2332 infected groups.

**Additional file 5: Table S4** Lists KEGG pathway enrichment of up-regulated and down-regulated mRNAs in GSWW and VR2332 infected groups.

**Additional file 6: Table S5** Lists KEGG pathway enrichment of mRNAs co-localized with differently expressed lncRNAs in GSWW and VR2332 infected groups.

**Additional file 7: Table S6** Lists KEGG pathway enrichment of mRNAs co-expressed with differently expressed lncRNAs in GSWW and VR2332 infected groups.

**Additional file 8: Table S7** Lists Real-time primers and siRNAs used in this study.

## Data Availability

Accession codes for GSWW_2015_full_GENOME sequence: GenBank No. KX767091. Accession codes for RNA Sequencing: BioProject ID PRJNA716767. To whom requests for materials should be addressed (email: luzengjun@caas.cn).

## Abbreviations

PRRSV: Porcine reproductive and respiratory syndrome virus
lncRNAs: Long non-coding RNAs
ISGs: Interferon-stimulated genes
siRNAs: Short interfering RNAs
MX1: MX Dynamin Like GTPase 1
CXCL10: C-X-C Motif Chemokine Ligand 10
RSAD2: Radical S-Adenosyl Methionine Domain Containing 2
