# Supplementary material for: The long non-coding RNA LNC_000397 negatively regulates PRRSV replication through induction of interferon-stimulated genes
Source: Virol J. 2022 Mar 5;19:40. doi: 10.1186/s12985-022-01761-x (PMC8897765; doi:10.1186/s12985-022-01761-x)
Supplement: Supplementary file 1 — Additional file 1: Figure S1 RT-qPCR results of eight differentially expressed genes after PRRSV infection by GSWW and VR2332 at 24 hpi. Total RNA was extracted by Trizol, and the first strand cDNA was synthesized using reverse transcriptase kit. Bar represents the mean of three samples. Expression levels were normalized to GAPDH. Figure S2 shows GO enrichment of up-regulated mRNAs in GSWW (a) and VR2332 (b) infected groups. Figure S3 shows Scatter plots of KEGG pathway enrichment statistics. Top 20 statistics of pathways, enrichment in the KEGG database for up-regulated mRNAs in GSWW infection group. Figure S4 shows Scatter plots of KEGG pathway enrichment statistics. Top 20 statistics of pathways, enrichment in the KEGG database for up-regulated mRNAs in VR2332 infection group. Figure S5 shows Scatter plots of KEGG pathway enrichment statistics. Top 20 statistics of pathways, enrichment in the KEGG database for down-regulated mRNAs in VR2332 infection group. [file 12985_2022_1761_MOESM1_ESM.docx]

Supplementary information

A novel long non-coding RNA LNC_000397 suppresses PRRSV replication in porcine alveolar macrophages

Jing Zhang, Lipeng Gan, Pu Sun, Zhijia Wang, Kun Li, Dong Li, Yimei Cao, Yuanfang Fu, Pinghua Li, Xingwen Bai, Xueqing Ma, Huifang Bao, Yingli Chen, Delong Wei, Wei Liu, Zaixin Liu*, Zengjun Lu*

*State Key Laboratory of Veterinary Etiological Biology, OIE/National Foot-and-Mouth Disease Reference Laboratory of China, Lanzhou Veterinary Research Institute, Chinese Academy of Agricultural Sciences, Lanzhou 730046, China*

*Corresponding author.

Tel: +86-931-8343390; Fax: +86-931-8340977.

E-mail: [liuzaixin@](mailto:liuzaixin3@)caas.cn; [luzengjun@caas.c](mailto:luzengjun@caas.c)n

Corresponding address: Lanzhou Veterinary Research Institute, Chinese Academy of Agricultural Sciences, Xujiaping No.1, Yanchangpu, Lanzhou, Gansu 730046, China

Figure S1.


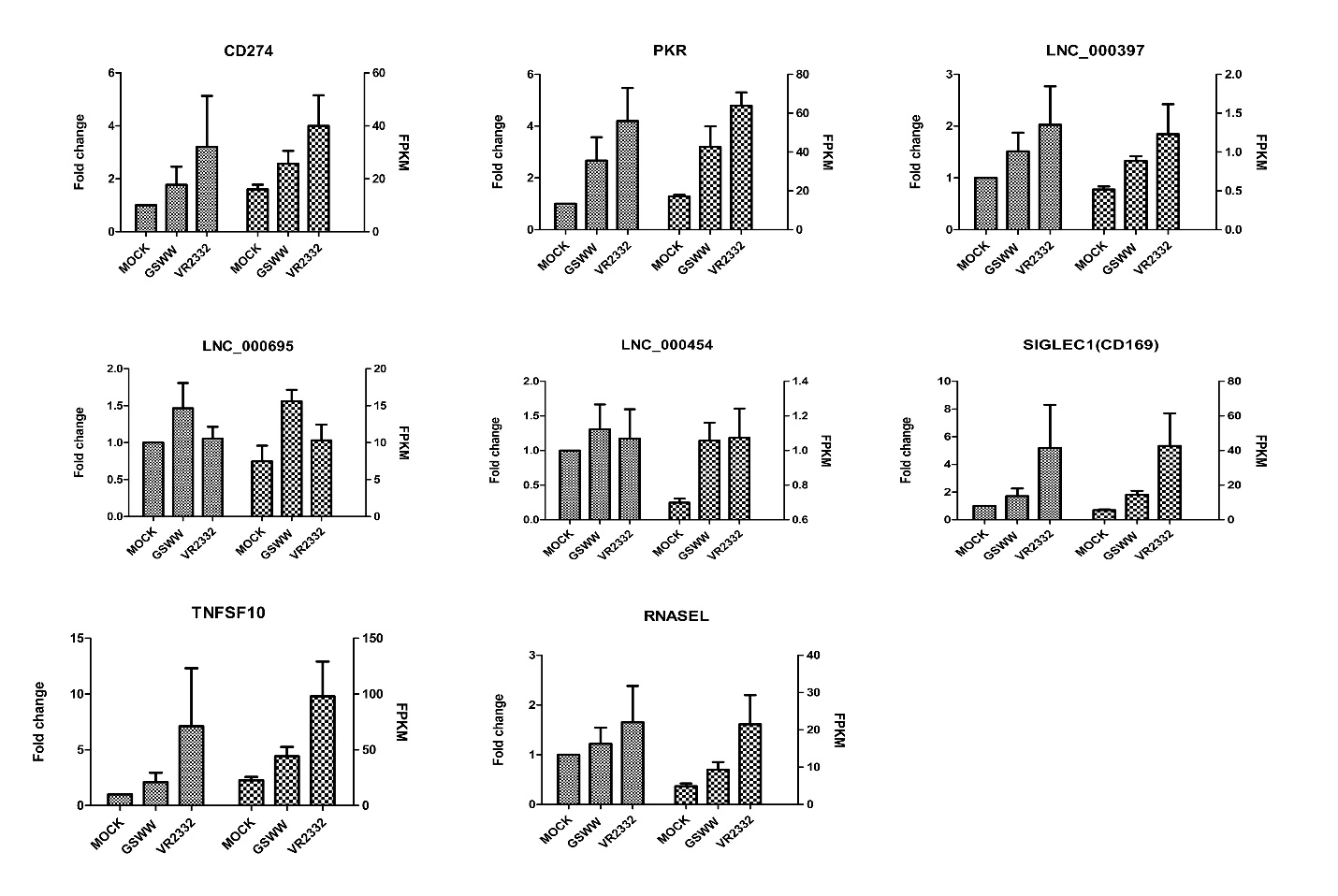


Supplementary Figure S1. RT-qPCR results of eight differentially expressed genes after PRRSV infection by GSWW and VR2332 at 24 hpi. Total RNA was extracted by Trizol, and the first strand cDNA was synthesized using reverse transcriptase kit. Bar represents the mean of three samples. Expression levels were normalized to GAPDH.

Figure S2.


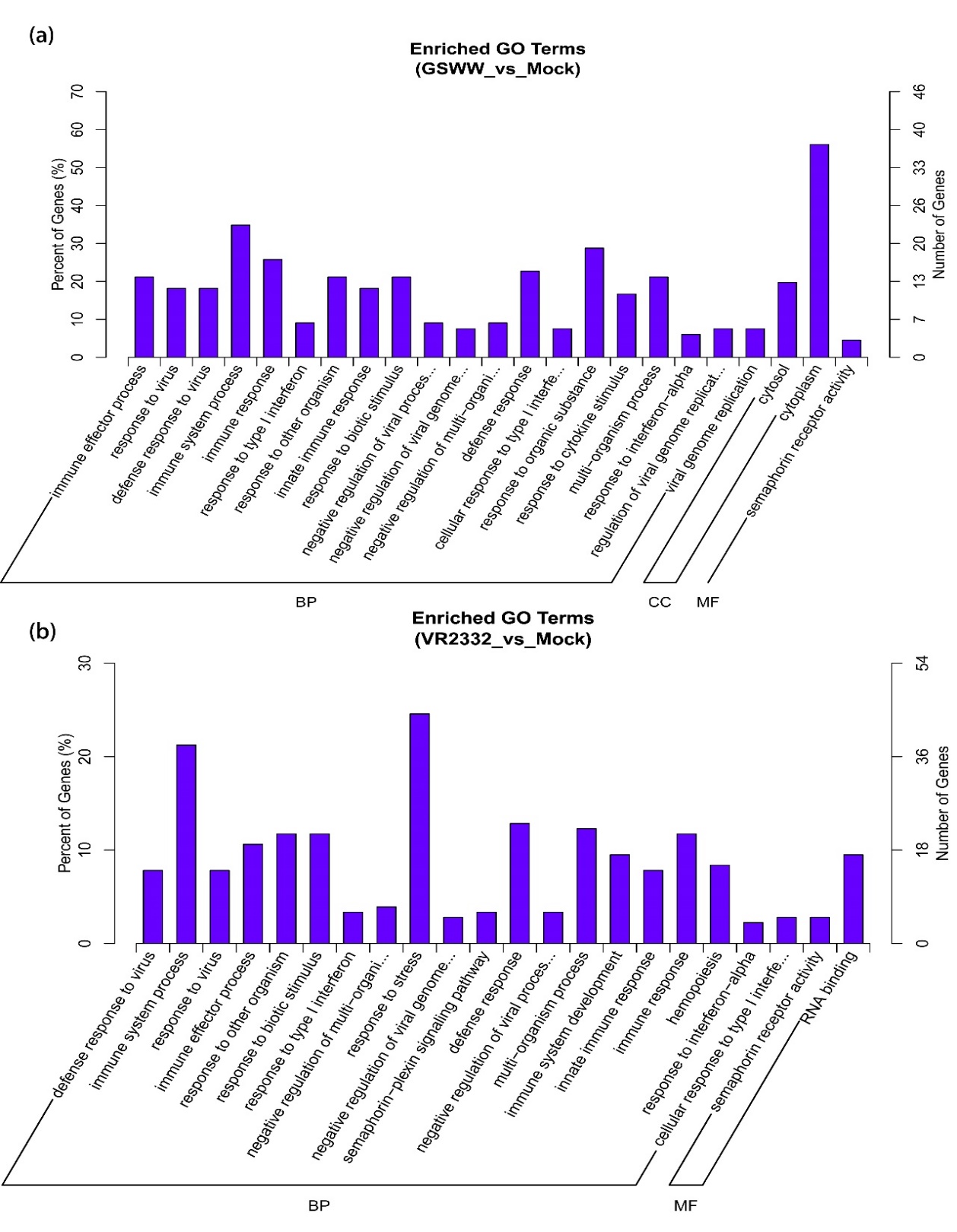


Supplementary Figure S2 shows GO enrichment of up-regulated mRNAs in GSWW (a) and VR2332 (b) infected groups.

Figure S3.


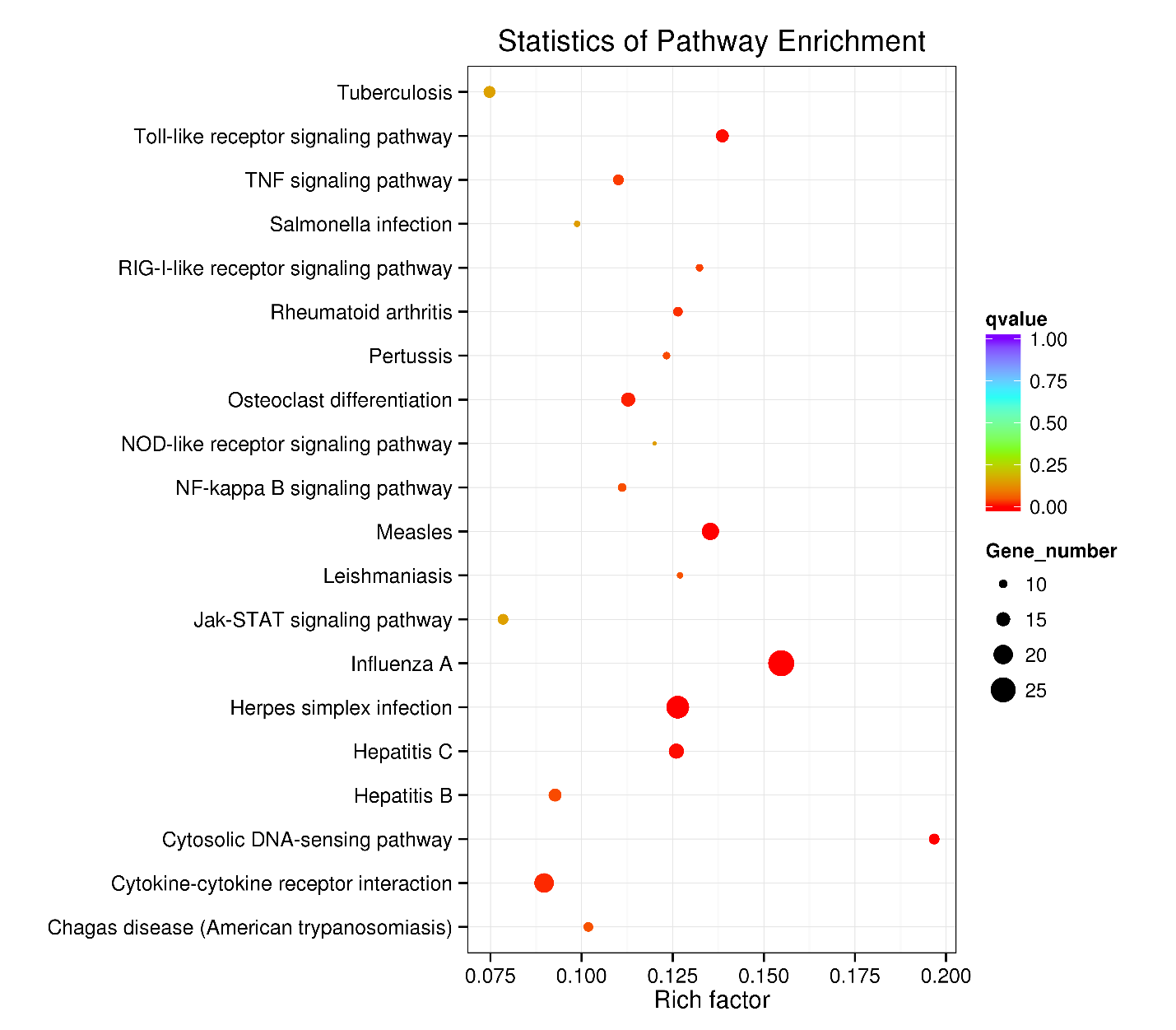


Supplementary Figure S3 shows Scatter plots of KEGG pathway enrichment statistics. Top 20 statistics of pathways, enrichment in the KEGG database for up-regulated mRNAs in GSWW infection group.

Figure S4.


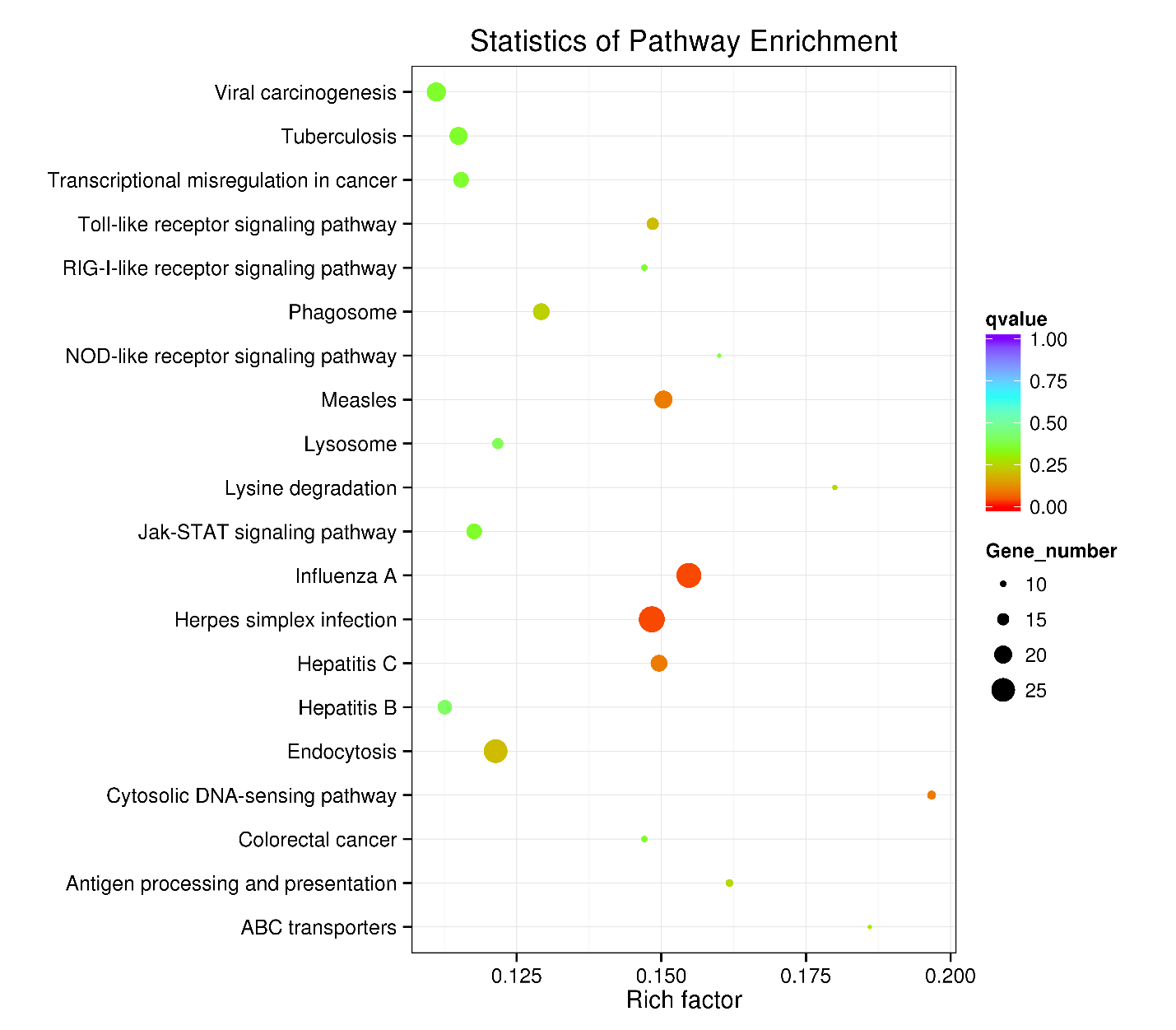


Supplementary Figure S4 shows Scatter plots of KEGG pathway enrichment statistics. Top 20 statistics of pathways, enrichment in the KEGG database for up-regulated mRNAs in VR2332 infection group.

Figure S5.


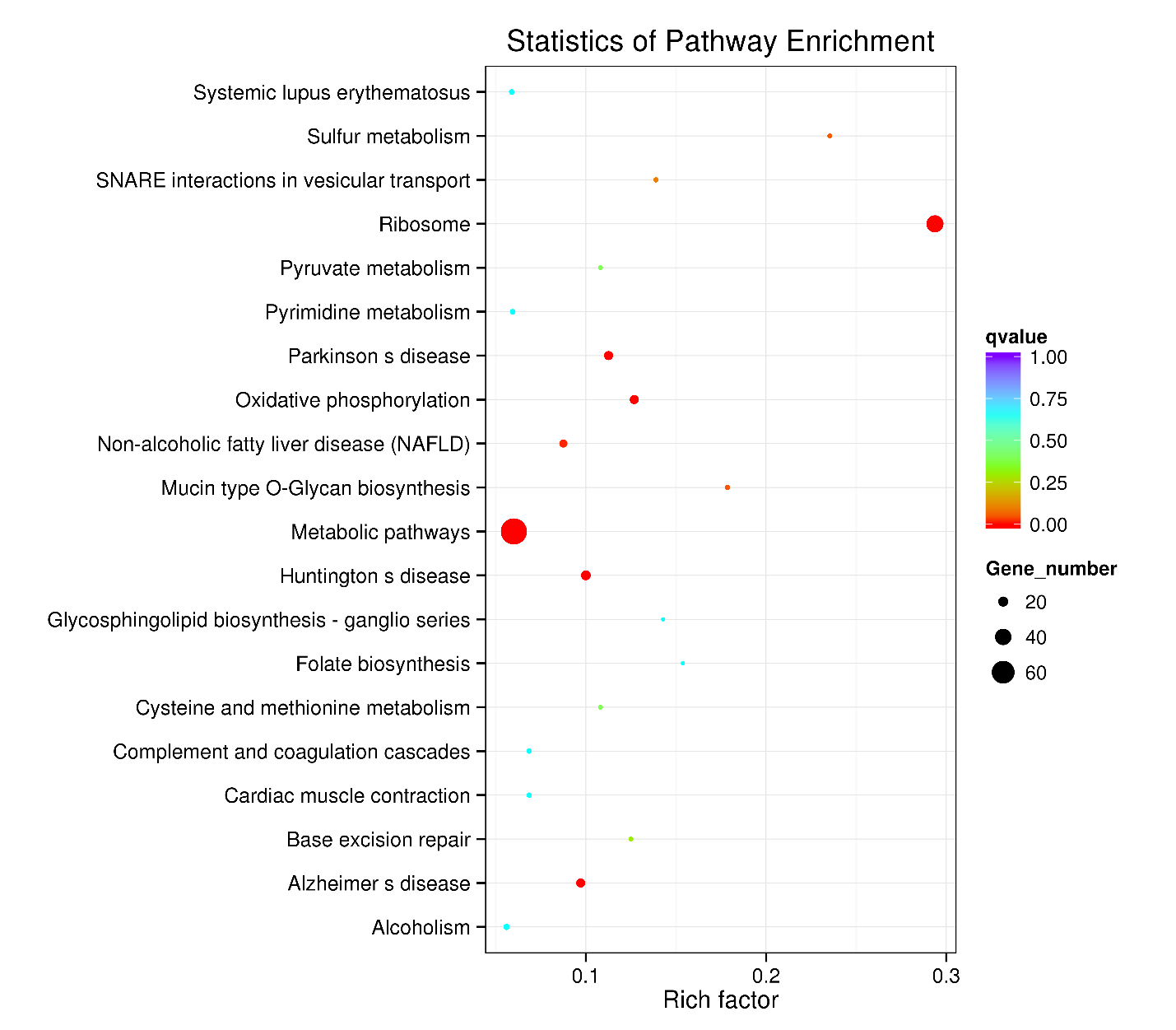


Supplementary Figure S5 shows Scatter plots of KEGG pathway enrichment statistics. Top 20 statistics of pathways, enrichment in the KEGG database for down-regulated mRNAs in VR2332 infection group.

Supplementary table S1 lists differently expressed mRNAs upon GSWW and VR2332 infection compared to Mock infection.

Supplementary table S2 lists differently expressed lncRNAs upon GSWW and VR2332 infection compared to Mock infection.

Supplementary table S3 lists GO enrichment of up-regulated and down-regulated mRNAs in GSWW and VR2332 infected groups.

Supplementary table S4 lists KEGG pathway enrichment of up-regulated and down-regulated mRNAs in GSWW and VR2332 infected groups.

Supplementary table S5 lists KEGG pathway enrichment of mRNAs co-localized with differently expressed lncRNAs in GSWW and VR2332 infected groups.

Supplementary table S6 lists KEGG pathway enrichment of mRNAs co-expressed with differently expressed lncRNAs in GSWW and VR2332 infected groups.

Supplementary table S7 lists Real-time primers ans siRNAs used in this study.
